# Electrocardiogram-based deep learning algorithm for the screening of obstructive coronary artery disease

**DOI:** 10.1186/s12872-023-03326-4

**Published:** 2023-06-07

**Authors:** Seong Huan Choi, Hyun-Gye Lee, Sang-Don Park, Jang-Whan Bae, Woojoo Lee, Mi-Sook Kim, Tae-Hun Kim, Won Kyung Lee

**Affiliations:** 1 Department of Cardiology, School of Medicine, Inha University Hospital, Inha University, Incheon, Korea; 2 School of Medicine, Inha University, Incheon, Korea; 3 Division of Cardiology, Department of Internal Medicine, Chungbuk National University College of Medicine, Cheongju, Korea; 4 Department of Public Health Sciences, Graduate School of Public Health, Seoul National University, Seoul, Korea; 5 Division of Clinical Epidemiology, Medical Research Collaborating Center, Biomedical Research Institution, Seoul National University Hospital, Seoul, Korea; 6 Department of Artificial Intelligence, Inha University, Incheon, Korea; 7 Department of Prevention and Management, School of Medicine, Inha University Hospital, Inha University, 27 Inhang-Ro, Jung-Gu, Incheon, Korea

**Keywords:** Obstructive coronary artery disease, Acute myocardial infarction, Deep learning, Convolutional neural network, Electrocardiogram

## Abstract

**Background:**

Information on electrocardiogram (ECG) has not been quantified in obstructive coronary artery disease (ObCAD), despite the deep learning (DL) algorithm being proposed as an effective diagnostic tool for acute myocardial infarction (AMI). Therefore, this study adopted a DL algorithm to suggest the screening of ObCAD from ECG.

**Methods:**

ECG voltage-time traces within a week from coronary angiography (CAG) were extracted for the patients who received CAG for suspected CAD in a single tertiary hospital from 2008 to 2020. After separating the AMI group, those were classified into ObCAD and non-ObCAD groups based on the CAG results. A DL-based model adopting ResNet was built to extract information from ECG data in the patients with ObCAD relative to those with non-ObCAD, and compared the performance with AMI. Moreover, subgroup analysis was conducted using ECG patterns of computer-assisted ECG interpretation.

**Results:**

The DL model demonstrated modest performance in suggesting the probability of ObCAD but excellent performance in detecting AMI. The AUC of the ObCAD model adopting 1D ResNet was 0.693 and 0.923 in detecting AMI. The accuracy, sensitivity, specificity, and F1 score of the DL model for screening ObCAD were 0.638, 0.639, 0.636, and 0.634, respectively, while the figures were up to 0.885, 0.769, 0.921, and 0.758 for detecting AMI, respectively. Subgroup analysis showed that the difference between normal and abnormal/borderline ECG groups was not notable.

**Conclusions:**

ECG-based DL model showed fair performance for assessing ObCAD and it may serve as an adjunct to the pre-test probability in patients with suspected ObCAD during the initial evaluation. With further refinement and evaluation, ECG coupled with the DL algorithm may provide potential front-line screening support in the resource-intensive diagnostic pathways.

## Introduction

Electrocardiogram (ECG) is a mainstay in the diagnosis of acute myocardial infarction (AMI) with biomarkers: a rise in troponin with at least one value > 99th percent upper reference limit [1–3]. In emergency departments, an AMI is classified into an ST-segment elevation myocardial infarction (STEMI), which requires emergent reperfusion treatment, and non-STEMI, which needs early intervention or conservative management, according to the ECG manifestation [4]. It also provides information, including the duration, extent, and location of the myocardial infarction, although initial ECG is often not diagnostic and serial ECG is required [5, 6].

On the other hand, the resting 12-lead ECG has not been critical for screening and diagnosing coronary artery disease (CAD) in patients with stable chest pain and suspected angina pectoris, but it remains an indispensable component of an initial evaluation [2, 3]. According to the JACC guidelines, the probability of obstructive CAD (ObCAD) should be considered when providing diagnostic tests to those with stable chest pain [2]. Basic tests (laboratory biochemical testing, a resting ECG, echocardiography, and possible ambulatory ECG monitoring) in patients with suspected CAD were used to determine who should be screened or may be deferred, after the pre-test probability was estimated using the age, sex, and symptoms, according to the ESC guidelines [3]. On the other hand, the probability of ObCAD could not be quantified after acquiring the resting ECG and it has been undetermined how much information from a resting ECG could contribute to the clinical decision to proceed with ObCAD diagnostic tests.

Recently, deep learning (DL) algorithms have demonstrated good to excellent performance in detecting AMI using ECG signals. A review study revealed the accuracy ranged from 80.6 to 99.9% for normal versus AMI detection in 11 DL-based models, and the other review study showed it from 83 to 99.9% in six DL models [7, 8]. Although previous studies showed the potential of DL approaches in detecting AMI and other cardiovascular diseases [7–10], few studies have used DL algorithms to utilize the information on ECG in patient screening of ObCAD [11, 12]. It may be due to differences in the pathophysiology and ECG changes between ObCAD and AMI although both belong to the CAD category. ObCAD is the progressive narrowing of coronary arteries, usually caused by atherosclerosis with no ECG characteristics or subtle, whereas AMI resulted from acute obstruction of coronary artery commonly by thrombosis, resulting in myocardial necrosis and more obvious ECG change. Therefore, in a previous study, the common DL model showed completely different discrimination (0.973 and 0.566 in AUC) between two subgroups separated by AMI and ischemia at diagnosis [13].

Therefore, a DL-based model was developed using ECG to suggest the need for further investigation for ObCAD in patients with chest pain and suspected ObCAD. Moreover, the performance of the model was evaluated to test the validity for screening ObCAD and compare it with that of AMI.

## Materials and methods

### Data sources and study population

This investigation was a retrospective observational study of consecutive patients who received coronary angiography (CAG) for suspected CAD in a single tertiary hospital. The patients were eligible if they were aged 18 years or older and underwent CAG due to suspected ObCAD from October 27, 2008, to August 21, 2020, at the Inha University Hospital, which was a university teaching hospital in Incheon, which had a population of 2,922,121 inhabitants in 2020, in South Korea. It has Regional Cardiocerebrovascular Centers (RCCVCs), established by the Ministry of Health and Welfare in the Incheon district.

### Data generation

The digital, standard 10-second, 12-lead ECG was acquired in the supine position during the study period. ECG was acquired at a sampling rate of 500 Hz using a GE-Marquette ECG machine (Marquette, WI, USA), and the raw data on ECG were extracted from the MUSE data management system (GE Healthcare, USA).

**Fig. 1:**
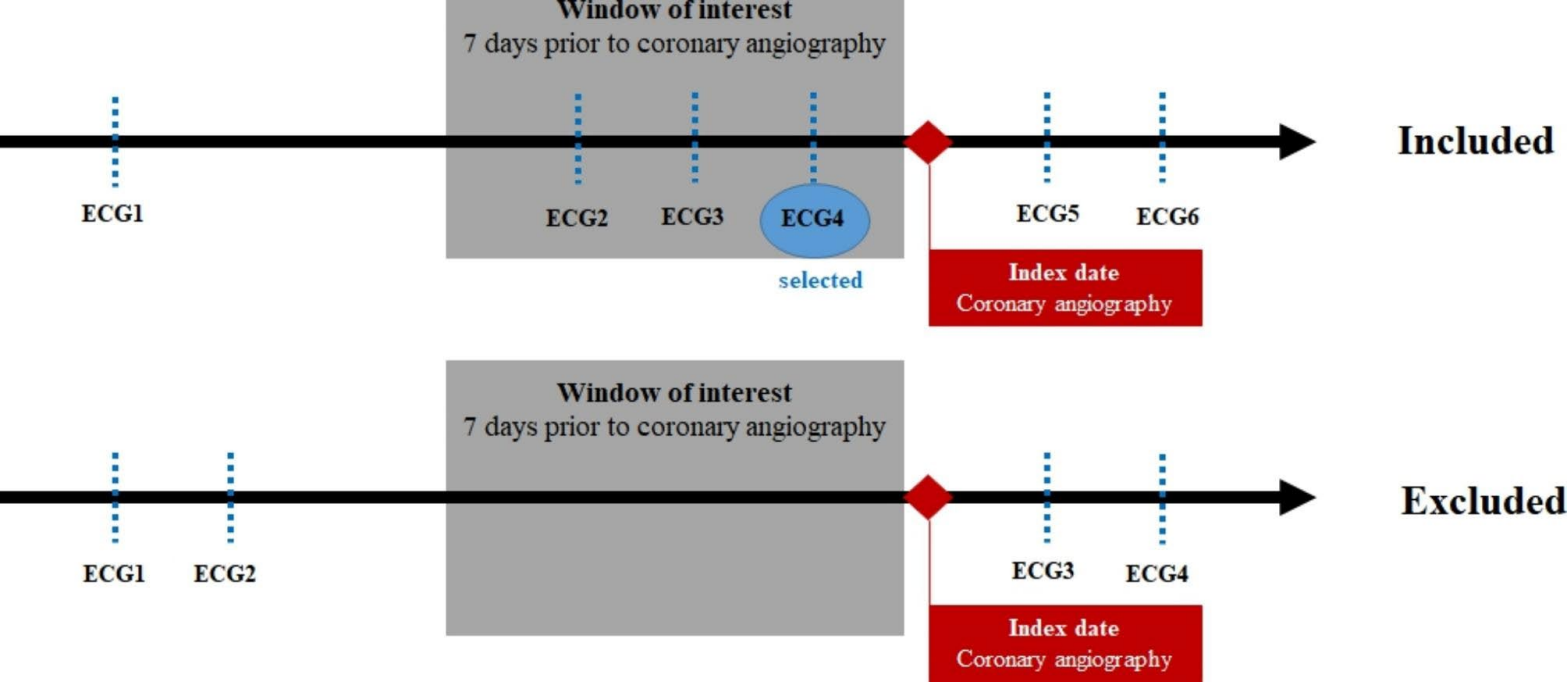
Timeline and time window of the electrocardiogram and coronary angiography data

The ECG was selected in a window of interest for each participant for analysis because most of the study participants had multiple ECG records over the study period. The index date and time were defined as the date and time when CAG started, and the window of interest was defined as the preceding seven days before the index date. The ECG within a week before CAG was selected for analysis. This window of interest was chosen under the assumption that the ECG within a week would have clues on the quantitative coronary angiography (QCA) stenosis in patients with ObCAD. Any patient who did not have an ECG in the window of interest was excluded. If patients had multiple ECGs in the preceding seven days, the most recent ECG, for which physicians decided whether to provide diagnostic CAG, was selected. Figure 1 illustrated the timeline and time window of the ECG and CAG data. Sensitivity analysis was conducted with the earliest ECG in the window period.

For the comparison of data distribution among the non-ObCAD, ObCAD, and AMI groups, 31 electrocardiographic patterns and eight quantitative ECG measurements were extracted from the ECG data and summarized for each group [14]. The eight ECG measurements included the QRS duration, QT, QTc, PR interval, ventricular rate, and the P-, Q- and T-wave axes. The ECG patterns were parsed and classified from the structured statements of computer-assisted ECG interpretation based on the standard key phrases in the MUSE data management system; the 31 patterns are listed in Table 1. An ECG was classified into two groups: normal and abnormal/borderline. The ECG was labeled as ‘normal’ if there was no abnormality in the interpretation and ‘abnormal’ or ‘borderline’ if the pattern included at least one diagnostic abnormality. Sex and age were also extracted from the electronic medical record and merged with CAG reports.

### Classification

The CAG reports were extracted from the electronic medical system. The dataset was divided into acute myocardial infarction and suspected angina pectoris to compare the performances between the DL models of ObCAD and AMI. After excluding the patients finally diagnosed with AMI, ObCAD was defined as the stenosis ≥ 50% luminal narrowing of any major vessel in QCA, and non-ObCAD as < 50%; it was defined to identify patients whose QCA showed significant stenosis more than 50% and those who could have benefited from further non-invasive diagnostic tests and CAG [15–17].

### ECG-based DL algorithm

**Fig. 2:**
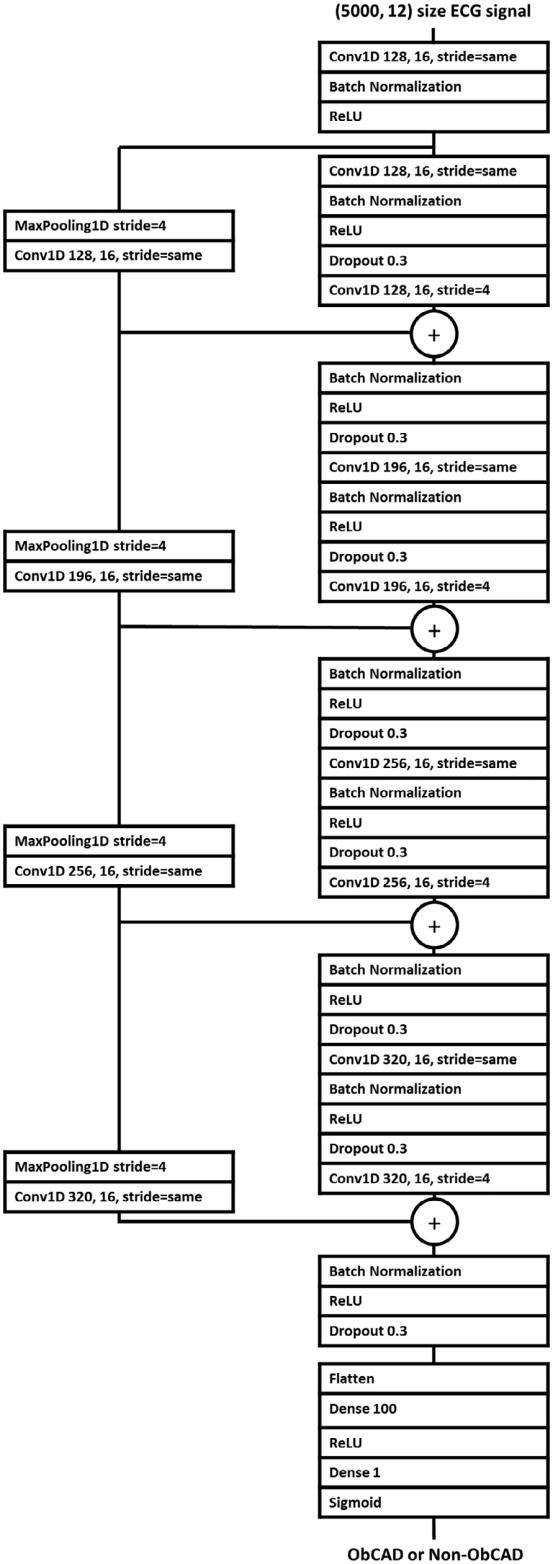
Proposed architecture of the deep learning-based electrocardiogram model

1D ResNet was suggested as a useful architecture for classifying ECG [18–20]. The model was implemented using Keras (version 2.0) with Tensorflow (Google; Mountain View, CA, USA). The proposed architecture of the ECG DL model using ResNet (ECGNET) was illustrated in Fig. 2. The performance of the proposed model was compared with those of four other models adopting machine learning (ML) and DL algorithms (logistic regression [LR], random forest [RF], long short term memory [LSTM], and transformer).

### Models comparison

A resting ECG consists of 12 vectors with 5,000 dimensions per sample, which is a very large input dimension compared to the number of training samples. Therefore, raw ECG signal is not suitable for use in traditional ML classifiers such as LR and RF. We used a fast Fourier transform (FFT) to extract the 10–100 Hz range from each lead in 10 Hz intervals to transform it into a dimensionality suitable for use with traditional ML classifiers. This was finally transformed into a vector with a total of 120 dimensions for the 12 leads. Then, ECG transformed by FFT was used as input for LR and RF. Using grid search, we set the hyperparameters of the RF model: number of estimators, minimum number of samples required to split, and minimum number of samples required to be at a leaf node to 100, 2, and 2, respectively. Bi-LSTM used L2 kernel regularizer, dropout 0.2, and ReLU activation function. In this study, we did not use the Transformer Encoder immediately before the Fully Connected (FC) Layer as in the study for ECG Arrhythmia Classification to identify the ECG pattern of ObCAD, but adopted the method of analyzing the time series with LSTM by determining and weighting the importance through self-attention to the extracted convolutional neural network (CNN) features. The ECG signal is compressed to a size of (256,64) through 1D CNN, and the same size (256,64) data is extracted through MultiHeadAttention. Finally, it was classified by sequentially passing the LSTM layer, FC layer, and sigmoid function.

The eight physical leads (lead I, II, and V1-6) were used in the 12-lead ECG because the four augmented leads (lead III, aVR, aVL, and aVF) were produced by a linear function of leads I and II. The horizontal long axis (10 s at 500 Hz) was denoted as the temporal axis to extract the morphological and temporal features, while the short axis (eight physical leads) represented the spatial axis to use layers from all the leads.

The ECG DL models were cross-validated using stratified 5-fold to estimate the average predictive performance. The validation set was randomly selected from the train set and the dataset was divided into train, validation, and test sets with an 3:1:1 ratio. The training dataset was used to train the DL model and optimize the hyperparameters with a validation dataset. The test dataset containing the remaining patients not used in the training or validation was used to evaluate the performance of the ECG-based DL algorithm. A diagnostic threshold was selected using the area under the curve (AUC) of the receiving operating characteristic (ROC) curve for the validation set. The threshold was then applied to the test dataset to calculate the precision, recall, accuracy, and F1 score.

The hyperparameters were compared among the following options: ResNet with the residual blocks (2, 4, and 8), kernel size (16, 32, and 64), batch size (4, 8, 16, 32, and 64), initial learning rate (0.1, 0.01, 0.001, and 0.0001), optimization algorithms (SGD, ADAM), and dropout rate (0, 0.3, and 0.8). The best hyperparameters achieving the highest F1-score in the validation set were 4, 16, 8, 0.001, and 0.3, respectively, for residual blocks, kernel size, batch size, initial learning rate, and dropout rate with optimization algorithms of the Adam optimizer.

Subgroup analysis was performed to get insight into whether the performance depended on the ECG diagnostic abnormalities in the interpretation which were provided by the GE-Marquette ECG machine.

## Results

**Fig. 3:**
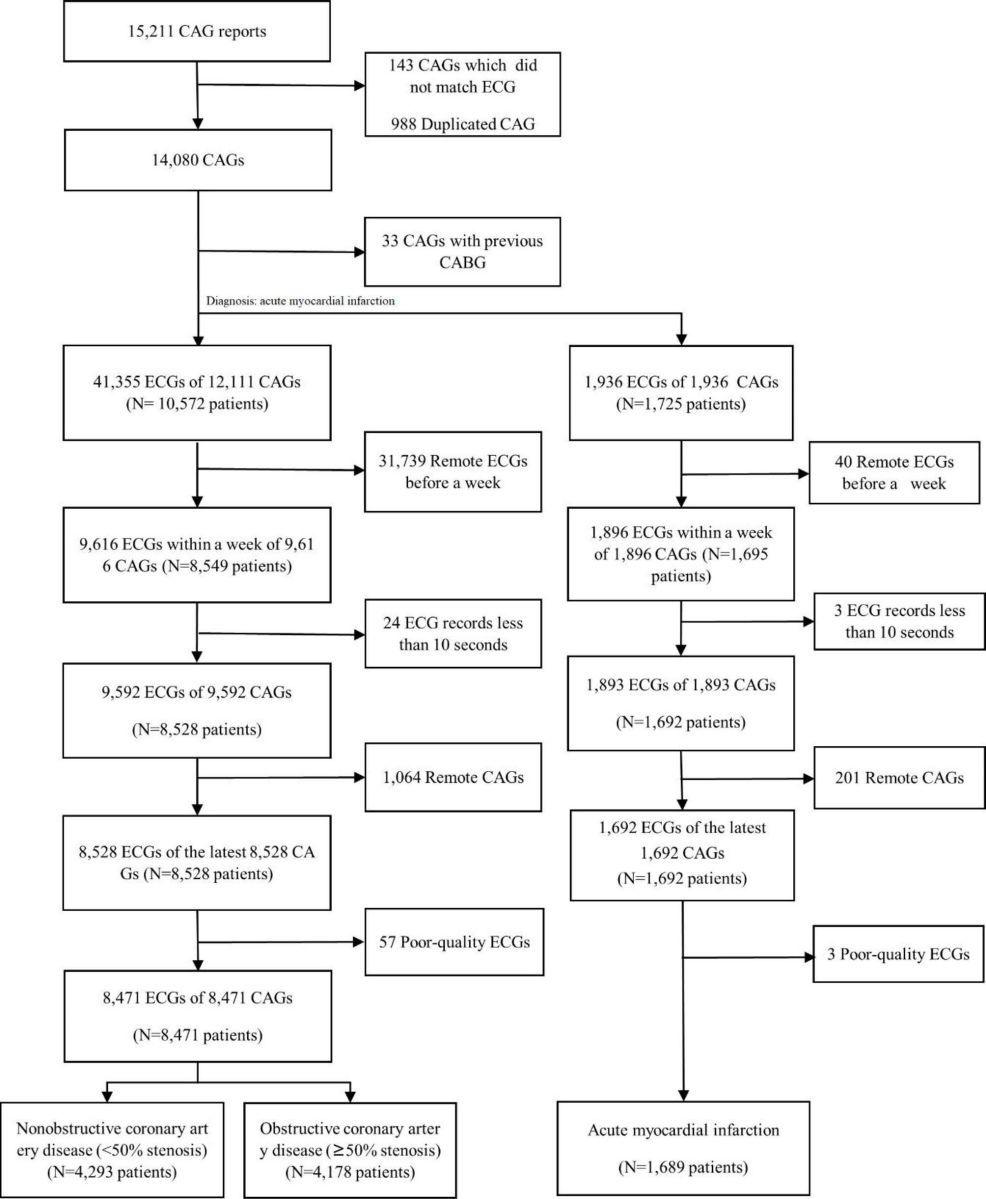
Flowchart of the data used in the study

After excluding the CAG unmatched with ECG and duplicated reports, 14,080 CAG reports were included with matched 41,355 ECG data (Fig. 3). Among all CAG reports, 1,689 patients diagnosed with AMI were finally selected for the DL-based model in detecting AMI. After excluding the ECG records with less than 10 s and ECGs recorded before a time window, the latest 9,592 ECG records were selected for 9,592 CAGs. A single CAG per patient was selected, and the most recent ECG was sampled, while the previous ECGs were excluded; it was assumed that the cardiologist decided the provision of CAG depending on the last ECG. Among the 9,592 ECGs, 1,064 ECG was excluded due to the patients who underwent multiple CAGs and 57 ECG records were of poor quality and were removed from the corresponding number of subjects. Of the remaining 8,471 patients, 4,293 and 4,178 patients were classified in the non-ObCAD group and ObCAD group, respectively, based on the findings from CAG reports.

Those in the ObCAD group were likely to be older than those in the non-ObCAD group (Table 1). Moreover, those with stenosis more than 50% were more likely to be male than those with stenosis less than 50%, but the majority of those enrolled were male in both groups. The traditional computer-assisted measurements and interpretations of ECG were suggested for each group in Table 1. The QRS duration and QT, QTc interval tended to be longer in the ObCAD group and AMI group than the non-ObCAD group. Traditional computer-assisted interpretation was ‘normal’ in 31.7% of the non-ObCAD group, while 20.6% in the ObCAD group. Moreover, it showed findings suggestive of AMI in 43.5% of patients finally diagnosed with AMI, while only in 2.4% of patients in the non-ObCAD group. On the other hand, traditional interpretation could not find the characteristics suggestive of ischemia as much as AMI, and the difference between the ObCAD and non-ObCAD group was not as definite as the difference between the AMI group and non-ObCAD group: 11.1% in the non-ObCAD group vs. 15.0% in the ObCAD group.

**Table 1:** Characteristics of the study population and data distribution

	All participants	Nonobstructive coronary artery disease (stenosis < 50%)	Obstructive coronary artery disease (stenosis ≥ 50%)	Acute myocardial infarction	p-value
	N = 10,160	N = 4,293	N = 4,178	N = 1,689	
**Demographic characteristics**					
Age (years)	63.6 (12.7)	60.9 (13.0)	66.7 (11.4)	62.2 (13.5)	< 0.001
Female (%)	29.3%	44.9%	31.5%	20.4%	< 0.001
**Measurement of Electrocardiographic features**					
QRS duration (ms)	97.6 (18.6)	95.9 (18.2)	97.2 (18.4)	98.6 (20.0)	< 0.001
QT (ms)	406 (45)	402 (43)	405 (45)	409 (50)	< 0.001
QTc (ms)	446 (39)	439 (38)	442 (39)	451 (39)	< 0.001
PR interval (ms)	168 (28)	165 (27)	169 (28)	168 (30)	< 0.001
Ventricular rate (bpm)	75.1 (17.9)	74.1 (17.2)	74.1 (17.9)	76.0 (19.6)	< 0.001
P axis (˚)	52 (36–64)	52 (35–64)	52 (36–63)	53 (37–66)	0.009
R axis (˚)	31 (2–58)	35 (8–60)	27 (-1-55)	34 (-2-63)	< 0.001
T axis (˚)	49 (25–76)	44 (24–63)	51 (26–79)	65 (29–93)	< 0.001
**Computer-assisted interpretation**					
Normal	15.8%	31.7%	20.6%	5.6%	< 0.001
Left bundle branch block	1.6%	2.2%	1.6%	1.3%	0.029
Incomplete left bundle branch block	0.3%	0.3%	0.2%	0.4%	0.517
Right bundle branch block	7.0%	5.4%	6.9%	7.9%	< 0.001
Incomplete right bundle branch block	2.0%	1.3%	1.9%	2.5%	0.003
Complete heart block	0.2%	0.2%	0.1%	0.4%	0.046
Atrial fibrillation	5.5%	6.8%	5.1%	5.1%	0.001
Atrial flutter	0.3%	0.5%	0.4%	0.2%	0.390
Acute myocardial infarction	23.5%	2.4%	4.6%	43.5%	< 0.001
Left ventricular hypertrophy	8.3%	9.0%	11.7%	6.3%	< 0.001
Premature ventricular contractions	4.5%	3.7%	3.9%	5.2%	0.021
Premature atrial contractions	2.5%	2.3%	2.5%	2.6%	0.737
First-degree atrioventricular block	6.0%	4.5%	6.3%	6.6%	< 0.001
s-degree atrioventricular block	0.3%	0.1%	0.1%	0.5%	< 0.001
Fascicular block	2.0%	1.6%	1.7%	2.4%	0.099
Sinus bradycardia	15.5%	15.4%	17.1%	14.8%	0.031
Other bradycardia	0.6%	0.2%	0.2%	0.9%	< 0.001
Sinus tachycardia	7.4%	5.1%	5.5%	9.5%	< 0.001
Ventricular tachycardia	0.2%	0.0%	0.2%	0.2%	0.038
Supraventricular tachycardia	0.1%	0.0%	0.1%	0.1%	0.390
Prolonged QT	8.2%	7.7%	7.2%	8.9%	0.084
Pacemaker	0.5%	0.5%	0.5%	0.5%	0.995
Ischemia	15.1%	11.1%	15.0%	17.2%	< 0.001
Low QRS voltage	2.9%	1.5%	2.1%	4.0%	< 0.001
Intraventricular block	2.2%	0.6%	0.9%	3.6%	< 0.001
Prior infarct	24.6%	9.0%	21.9%	33.9%	< 0.001
Nonspecific T-wave abnormality	5.6%	8.2%	8.0%	3.0%	< 0.001
Nonspecific ST abnormality	3.9%	3.7%	5.0%	3.4%	0.003
Left axis deviation	7.5%	5.2%	6.7%	9.0%	< 0.001
Right axis deviation	0.5%	0.3%	0.2%	0.7%	0.012
Early repolarization	1.3%	2.0%	1.0%	1.1%	< 0.001

Table 2 lists the performances of the model for ObCAD and AMI. The AUC of the DL model in the test dataset was 0.693 for ObCAD, while it was 0.923 in detecting AMI. The accuracy, sensitivity, specificity, and F1 score of the proposed ECGNET model for screening ObCAD from ECGs were 0.638, 0.639, 0.636, and 0.634, respectively, in the test set. On the other hand, the figures were up to 0.885, 0.769, 0.921, and 0.758 for detecting AMI, respectively. By contrast, the performance was not notable between two subgroups classified by the traditional automated interpretation when the ObCAD dataset was divided into normal and abnormal/borderline ECG. The AUC was 0.716 and 0.728 for normal and abnormal/borderline ECG, respectively. When the model for ObCAD was built with the earliest ECG in the window period, the performance did not change significantly from that of the current model with the most recent ECG (data not shown).

**Table 2:** Performance of the prediction models. Bold values denotes the best performance across the different algorithms for predicting ObCAD and AMI.

	AUC	Accuracy	Sensitivity	Specificity	Precision	F1-score
**Obstructive coronary artery disease**						
Logistic Regression	0.528	0.522	0.499	0.545	0.516	0.506
Bi-LSTM	0.515	0.515	0.490	0.539	0.508	0.498
Random Forest	0.523	0.524	0.497	0.550	0.518	0.507
Transformer	0.647	0.612	0.596	0.627	0.609	0.602
ECGNET	**0.693**	**0.638**	**0.639**	**0.636**	**0.632**	**0.634**
**Acute myocardial infarction**						
Logistic Regression	0.620	0.599	0.598	0.600	0.310	0.408
LSTM	0.692	0.690	0.604	0.715	0.398	0.475
Random Forest	0.515	0.770	0.038	0.991	0.575	0.072
Transformer	0.871	0.816	0.758	0.834	0.582	0.657
ECGNET	**0.923**	**0.885**	**0.769**	**0.921**	**0.749**	**0.758**
**Coronary artery disease subset by traditional computer-assisted ECG interpretation**						
Normal ECG	0.716	0.646	0.729	0.592	0.564	0.645
Abnormal/borderline ECG	0.728	0.679	0.728	0.622	0.689	0.675

LSTM, long short term memory; ECGNET, ECG 1D ResNet model

**Fig. 4:**
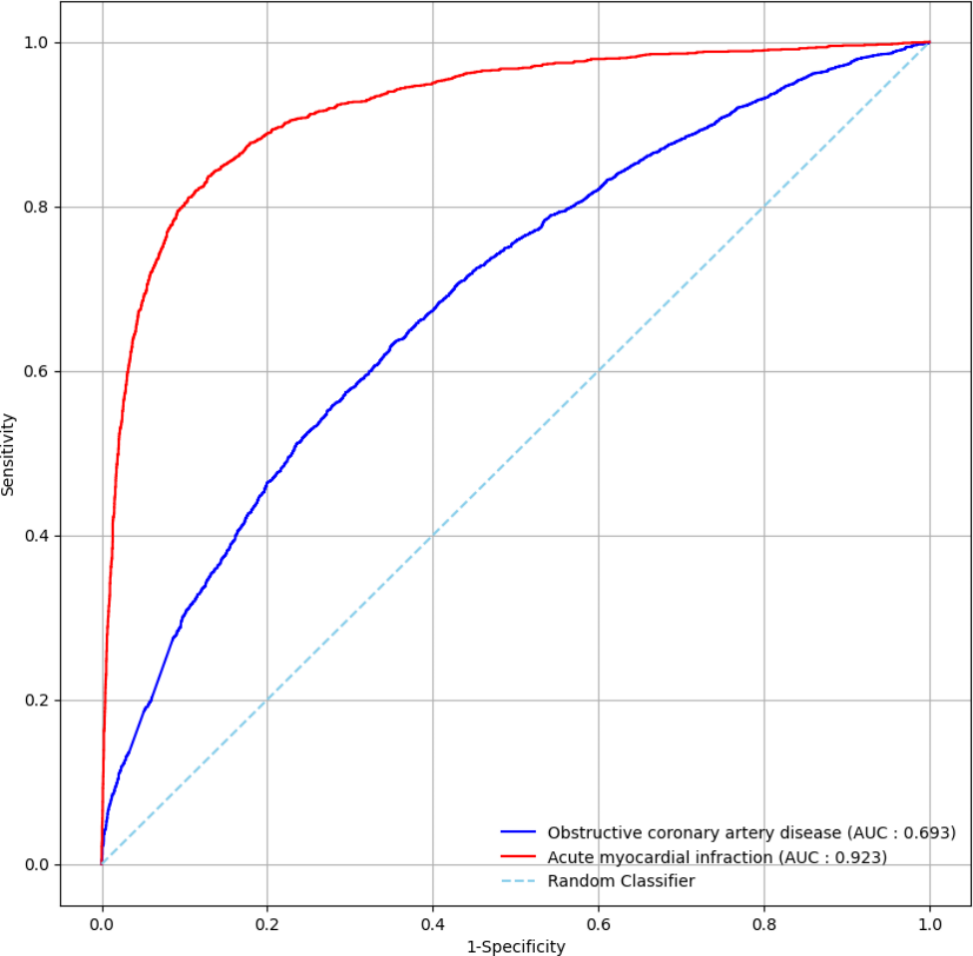
ROC curves for the developed model of obstructive coronary artery disease and acute myocardial infarction on the testing dataset ROC: receiver operating characteristic, AUC: area under the curve

Figure 4 presents the ROC curve of the model for ObCAD and AMI.

## Discussion

A DL-based model adopting ResNet was constructed to extract information from ECG voltage-time traces in the patients with ObCAD relative to those with non-ObCAD. The model demonstrated fair performance in suggesting the probability of ObCAD, while it showed good to excellent performance in detecting AMI. Whereas the good performance similar to previous research for detecting AMI was achieved by the current study, the model for screening ObCAD which had been little attempted showed that it was more complex and daunting task compared to AMI. Interestingly, the performance did not depend on whether it has pre-defined ECG features by the traditional computerized interpretation; the performance did not deteriorate in the normal ECG groups where the defined profiles of the ECG abnormality were not found.

Previous studies reported that DL algorithms were effective in detecting AMI. They reported excellent performance using various DL algorithms. A previous study revealed an AUC of 0.997 and 0.877 in STEMI and NSTEMI [21], respectively, and another suggested an AUC of 0.951 and 0.901 in STEMI and AMI, respectively [22]. Moreover, a review study suggested that DL-based models on the diagnosis of AMI have an accuracy above 95% [8]. On the other hand, most of the studies included were based on a small sample from the same open-source database (PTB-XL database) and focused on the experimental application of new algorithms [8, 23]; some studies showed good performance when CNN was adopted, and others suggested that the performance may be enhanced when other algorithms were added or applied: multi-lead residual neural network, fusion of features, multi-lead attention, bidirectional gated recurrent unit, variational autoencoder, and CNN coupled with LSTM/BLSTM network [7, 8, 21–25]. Therefore, a recent study reported that residual controversies or gaps in evidence exist on the value of ECG to identify acute coronary syndrome and has been conducted on the issues of validation in patients without ST-elevation, the role of ECG in identifying culprit lesions, P-wave abnormalities, Q-wave regression, and ST-deviation and resolution [26].

In contrast, the performance of the DL model for assessing stable ischemic heart disease has been rarely evaluated and only a few experiments have been attempted based on a small number of subjects. Recently, a systematic review suggested that it found two DL models for stable ischemic heart disease (IHD) in the review, which performed as well as six models for detecting AMI [7]. However, both studies on stable IHD used the same data from the PhysioNet database: seven CAD subjects from St. Petersburg Institute of Cardiology Technics 12-lead arrhythmia data [11, 12]. The analysis was based on only seven subjects diagnosed with CAD with hypertension. Moreover, among them, four patients had ECGs consistent with left ventricular hypertrophy (LVH), while the other five patients diagnosed with angina pectoris in the dataset were not included in their analysis [27]. Therefore, although the previous research suggested that the application of DL algorithms might be promising for detecting ObCAD, the performance might be over-estimated due to the distinct ECG characteristics of seven subjects with hypertension or LVH. Furthermore, the other studies also used relatively small sample sizes which limited generalization. They were conducted in the different settings: Gokhan Altan extracted the 21–24 h long-term ECGs of 60 subjects diagnosed with CAD from the Long-Term ST Database [28, 29] and Monappa Gundappa Poddar gathered ECG data of 64 male patients in the age group of 35–60 years who were previously healthy in India [30]. Therefore, a small sample size yielded less variation in the ECG data. The models should be evaluated on larger datasets with diversity to confirm the robustness in a real-world setting.

In this study, the performance of the DL model for screening ObCAD was modest compared to that of the model for diagnosing AMI. The ECG findings of AMI were more remarkable because AMI causes more irreversible tissue damage to the myocardium than ObCAD with stable chest pain. In contrast, the ECG perturbations tend to be subtle and have difficulty classifying ObCAD [8]. Although normal ECG does not exclude the possibility of angina pectoris, ECG could provide useful information on the screening of ObCAD [31, 32]. In the European Society of Cardiology guidelines, a resting ECG was first-line tested in patients with suspected CAD. The signs of myocardial ischemia were based mainly on the detection of repolarization abnormalities and indirectly on previous infarction or conduction abnormalities [3]. On the other hand, the findings varied significantly, depending on the duration, extent, and topography of ischemia and the presence of other underlying arrhythmias [33]. Furthermore, false-positive results were reported more commonly in the patients with LVH, electrolyte imbalance, use of digitalis, and intraventricular conduction abnormalities [33]. Therefore, it is more difficult to use ECG characteristics in an DL-based model to estimate the clinical probability of chronic CAD.

Current practice depends on the clinician’s interpretation on ECG when the patient with stable chest pain and suspected ObCAD is evaluated. Therefore, it is subjective according to the knowledge and experience of the clinician and requires time and effort in the field. It also could not be quantified and integrated into any quantitative estimation of risk stratification. In contrast, the DL-based ECG model could contribute to automated ECG interpretation and risk quantification. Furthermore, while ECG may be a poor predictor from the perspective of the human eye and computer-assisted ECG features provided by GE machine, ECG characteristics have been demonstrated to be related to the prediction of ObCAD [31, 32, 34–36]. Previous literature reported that the resting ECG could not properly predict ObCAD based on the cardiologist’s interpretation of the ST segment, T and Q wave: 51.5% of sensitivity and 66.1% of specificity [37]. Similarly, the ECG interpretation provided by GE showed lower sensitivity in this study; it found myocardial ischemia in only 15% of 4,178 patients with QCA stenosis, whereas 11% of the patients without QCA stenosis had ischemia in the interpretation (15.0% of sensitivity and 88.9% of specificity). However, other studies have suggested that multiple ECG variables in the transformed and multiadjusted models could be important predictors of ObCAD detection and mortality, and another recent studies suggested that heart rate variability and the Hilbert–Huang transformation of ECG may reveal the hidden information on the myocardial ischemia [32, 34, 35]. Therefore, if the DL-based ECG models are enhanced in sophisticated and innovative ways, they may help clinicians make better discrimination and decision for further diagnostic methods.

In this study, the proposed model adopting 1D ResNet was superior to other ML and DL models. It may be because LR and RF cannot reflect time series features when learning ECG signals. In particular, although RF improves generalization performance by preventing overfitting of decision trees through ensembles, we experimentally confirmed that it still tends to overfit in high-dimensional data [38]. To prevent this, we converted ECG signals to FFT, but the loss of frequency resolution and information and the loss of time series characteristics could not be completely solved [39]. Bi-LSTM considers time series characteristics, but its strength lies in learning long-term dependencies, making it less suitable for finding fine features with short-term periods [40]. Transformer Encoder’s Self-Attention was primarily used to learn global dependencies, which limited the 1D CNN’s ability to detect local patterns. In addition, the model complexity increases due to more parameters compared to the 1D CNN, which could lead to overfitting problems.

The main strength of this study was the inclusion of a contemporary population with suspected ObCAD and who received CAG. Therefore, there is little risk of misclassification because the classification was based on the CAG reports. Moreover, the number of the subjects was higher than those of previous experiments, which offers more variation in the ECG data and is close to the real world. Furthermore, the DL-based model could help more people receive an earlier diagnosis and treatment compared to traditional ECG interpretation and help to reduce unnecessary diagnostic tests in the current practice. Compared to traditional ECG interpretations, the DL-based model could lead to the earlier diagnosis of 58% more people with CAG and the earlier treatment of patients with stable chest pain and suspected ObCAD: 646 (15%) of the traditional interpretation vs. 3,050 (73%) of the DL model in 4,178 patients with QCA stenosis. Furthermore, 62% of the 4,293 patients with a low probability of ObCAD by the DL model could be excluded from unnecessary non-invasive diagnostic tests and CAG.

This study had some limitations. This was a retrospective study based on subjects who were not all comers with chest pain but had received CAG. Therefore, the enrolled subjects were the selected patients considered high-risk by a physician, which may limit generalization. Second, these results need to be transformed into applications in the future, such as being implemented in a prospective study, to confirm the performance. Third, although the DL algorithm showed good performance in automatically detecting AMI, it still showed fair accuracy and sensitivity in screening for ObCAD. Therefore, the results of this study should be interpreted with caution in clinical practice, and the DL-based ECG model should be innovatively improved for practical use in the future. Fourth, the characteristics of symptoms, which were critical in clinical practice, could not be covered by a better ECG interpretation. Finally, it is important to explore further the ECG components that contribute to the classification. Recently, efforts have been made to develop new technologies that could make machine-learning models interpretable or explainable.

## Conclusion

This study examined the possibility of adopting a DL algorithm to ECG for screening ObCAD and compared the performance of the ObCAD model with that of the AMI model. Although the model showed good to excellent performance in detecting AMI, it demonstrated only modest performance in suggesting the probability of ObCAD and required further enhancement. Nevertheless, information from ECG extracted by the DL algorithm may serve as an adjunct to an initial assessment by clinicians in addition to the pre-test probability. With further refinement and evaluation, ECG coupled with the DL algorithm may provide potential front-line screening support to assist clinicians in the resource-intensive diagnostic pathways.

## Data Availability

The Information security committee of Inha University Hospital will oversee any materials sharing processes. Requests for data should be addressed to the corresponding author.
